# Protocol for the CUPIDO trials; multicenter randomized controlled trials to assess the value of combining prolapse surgery and incontinence surgery in patients with genital prolapse and evident stress incontinence (CUPIDO I) and in patients with genital prolapse and occult stress incontinence (CUPIDO II)

**DOI:** 10.1186/1472-6874-10-16

**Published:** 2010-05-11

**Authors:** Annemarie van der Steen, Marinus van der Ploeg, Marcel GW Dijkgraaf, Huub van der Vaart, Jan-Paul WR Roovers

**Affiliations:** 1 Department of Obstetrics and Gynaecology, University Medical Centre Groningen, PO Box 30.001, 9700 RB, Groningen, the Netherlands; 2 Department of Obstetrics and Gynaecology, Martini Hospital, PO Box 30.033, 9700 RM, Groningen, the Netherlands; 3 Department of Epidemiology, Academic Medical Centre Amsterdam, University of Amsterdam, PO Box 22660, 1100 DD, Amsterdam, the Netherlands

## Abstract

**Background:**

About 40% of all patients with genital prolapse report stress-incontinence. In about half of the 60% patients that do not report stress-incontinence, occult urinary stress-incontinence can be detected. In these patients stress-incontinence is masked due to kinking or compression of the urethra by the prolapse.

In case surgical correction is indicated there are two strategies to manage patients with combined prolapse and (occult) stress incontinence. This strategy is either (i) a combination of prolapse surgery and stress-incontinence surgery or (ii) to correct the prolapse first and evaluate afterwards whether additional stress-incontinence surgery is indicated. The advantage of combining prolapse and stress-incontinence surgery is that only few patients report stress-incontinence following such combination. However, this combination has been associated with an increased risk on complications, of which the development of obstructive micturition symptoms, overactive bladder symptoms and bladder retention are the most important ones. Furthermore, combining two procedures may be unnecessary as performing only prolapse surgery may cure stress-incontinence

In the randomized CUPIDO trials both strategies are compared in patients with prolapse and evident stress incontinence (CUPIDO I trial) and in patients with prolapse and occult stress incontinence (CUPIDO II trial).

**Methods/Design:**

The CUPIDO trials are two multicenter randomized controlled trials in which women with stress urinary incontinence (SUI) or occult stress urinary incontinence (OSUI) are randomized to prolapse surgery combined with anti incontinence surgery (concomitant surgery) or to prolapse surgery only. Patients with at least stage 2 POP are eligible, women with evident SUI are randomized in CUPIDO I. Patients without SUI are eligible for CUPIDO II and will have urodynamic evaluation or a standardized redression test. Women with OSUI are randomized, women without OSUI are followed up but not randomized.

The primary outcome measure is absence of SUI twelve months after surgery. Furthermore, economic evaluations are conducted, and the effectiveness of urodynamic investigation is evaluated against a non-invasive way to determine SUI in women with POP.

A total of 450 women will be included in the study.

**Trial Registration:**

Trial registration http://www.trialregister.nl NTRR 1197 en 1070

## Background

About 40% of the women with pelvic organ prolapse (POP) also report stress urinary incontinence (SUI) [1]. If a woman with an indication for POP surgery does not report stress incontinence before surgery, the risk on de novo stress incontinence is reported to be 11-20% [2,3]. In the patients who leak during a pre-operative stress test during redression of the prolapse (defined as occult stress incontinence, OSUI) the risk on de novo stress incontinence may even be as high as 80% [4,5]. The underlying mechanism of occult stress incontinence is that the prolapse prevents stress incontinence by kinking or compression of the urethra. After the prolapse is surgically corrected the prolapse does not mask stress incontinence anymore.

In case of abdominal POP surgery, the CARE trial has shown that combining abdominal sacrocolpopexy with Burch colposuspension may decrease the risk of postoperative urinary incontinence without increasing other lower urinary tract symptoms [6]. Similar RCT's in patients undergoing vaginal POP surgery have not yet been published. As a consequence, it is still debatable whether vaginal POP surgery should be combined with a midurethral sling procedure or not.

Combining vaginal prolapse repair with anti-incontinence surgery showed to be an effective treatment for SUI in observational studies [7-10], but literature about possible side effects like obstructive voiding symptoms, overactive bladder symptoms or bladder retention is not consistent [8,10-15]. Concomitant surgery could lead to a higher success rate in curing SUI, but could also lead to overtreatment with the possible side effects of a midurethral sling procedure.

A randomized controlled trial published by Borstad et al showed that in case of co-existing complaints (evident SUI) prolapse repair gave a 3 months success rate in curing SUI of 29%. About 70% of patients still had SUI after surgery, but in part of these women the complaints were so minimal, that they declined the TVT procedure 3 months after the prolapse surgery [16].

In patients without evident SUI it is unclear how to predict the risk of postoperative SUI and how high this risk is. Possibly, this could be predicted using a stress test with redression of the prolapse during clinical examination or urodynamic investigation [17-19]. The patients that have a positive redression test (OSUI) may be at highest risk of developing postoperative SUI, and might benefit from combining prolapse surgery with a midurethral sling procedure.

We designed two randomized controlled trials to evaluate the clinical value of combining prolapse surgery and stress incontinence surgery. The trials are referred to as CUPIDO which means: Concomitant surgery and Urodynamic investigation in genital Prolapse and stress Incontinence. A Diagnostic study including Outcome evaluation. CUPIDO I is a trial that evaluates whether concomitant surgery leads to better results in patients with evident SUI than prolapse repair only.

CUPIDO II is a trial that evaluates concomitant surgery versus prolapse surgery only in patients with OSUI.

## Methods/Design

### CUPIDO I Study aims

The primary aim of the CUPIDO I study is to determine whether combined prolapse surgery and anti-incontinence surgery in patients with POP and SUI results in less SUI postoperatively than correcting only the prolapse. Secondary aim is to find out whether concomitant surgery leads to an increase of adverse events, like obstructive voiding symptoms and detrusor overactivity.

### Design

The CUPIDO I trial is a multicenter randomized controlled trial. All patients with POP and SUI will be randomized for concomitant surgery or prolapse surgery only. SUI is defined as a history of SUI at least once a week and/or a positive cough test on examination, without redression of the prolapse.

### Determination and identification of eligible patients

**Figure 1:**
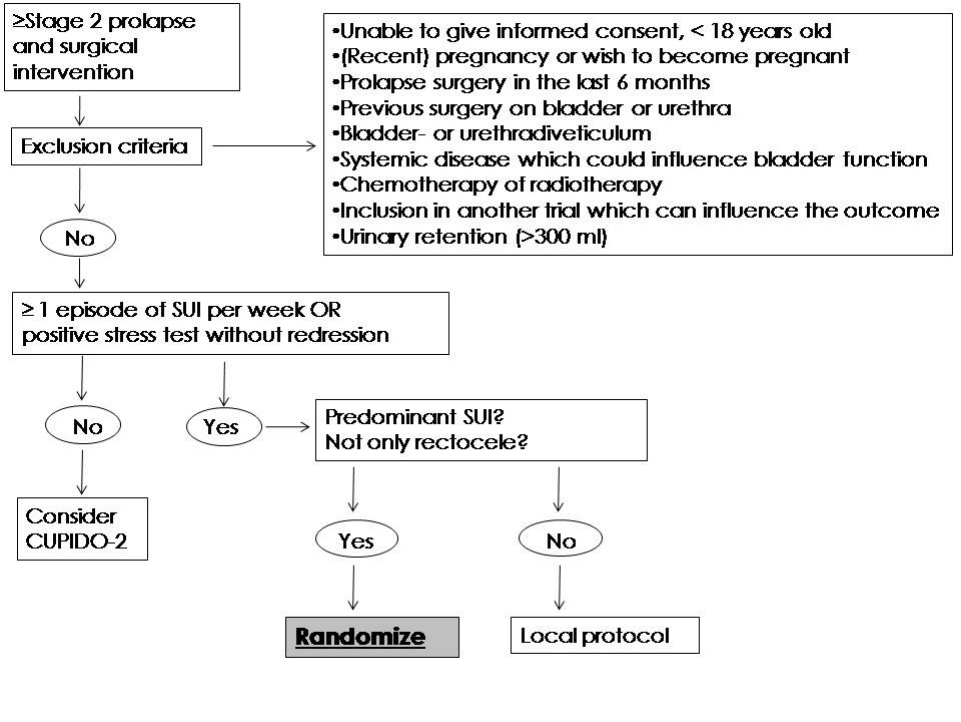
**Flowchart CUPIDO I**.

Eligible patients will be selected by gynaecologists of all participating hospitals in the Netherlands (Table 1). All women with at least stage 2 POP in whom operative correction of the prolapse is considered, will be asked for informed consent to participate in the trial (Figure 1). Women will be excluded in case of previous incontinence surgery, recent or current pregnancy, wish for pregnancy in the future, history of bladder- or urethrasurgery, systemic disease which can affect the bladder function, chemo- or radiotherapy for cancer, chronic retention of the bladder (> 300 ml), rectocele only, and participation in another intervention study which can have influence on the findings in this trial (Table 2).

**Table 1:** Participating hospitals in the Netherlands.

Academic Medical Center, Amsterdam
Alant Vrouw, Bilthoven
Deventer Hospital, Deventer
Martini Hospital, Groningen
Maxima Medical Center, Eindhoven
Medical Center Leeuwarden, Leeuwarden
Reinier de Graaf Groep, Delft
Sint Antonius Hospital, Nieuwegein
TweeSteden Hospital, Tilburg
Twenteborg Hospital, Almelo
University Medical Center, Groningen
University Medical Center, Nijmegen
University Medical Center, Maastricht
VieCuri Medical Center, Venlo

**Table 2:** Inclusion and exclusion criteria CUPIDO I.

Inclusion criteria	Exclusion criteria
≥ 18 years old	Inability to give informed consent
≥ stage 2 prolapse	(Recent or planned) pregnancy
Intended surgery for prolapse	Previous anti-incontinence surgery
	Recent prolapse surgery (<6 months)
	Previous surgery to bladder or urethra or diverticulum
	Systemic disease which could influence bladder function
	Planned chemo- or radiotherapy for neoplasm
	Participation in another study which could influence the results
	Chronic urinary retention
	Not predominant SUI (urge incontinence)
	Rectocele only

### Interventions

Patients are randomized for either combination surgery or for prolapse surgery only. Combination surgery is defined as a combination of prolapse surgery and stress incontinence surgery. Prolapse surgery can be a vaginal hysterectomy or sacrospinous fixation, Manchester Fothergill operation, anterior colporrhaphy or mesh implantation, posterior colporrhaphy or mesh implantation and enterocele repair. Anti-incontinence surgery can be a midurethral sling, either using the retropubic route or the trans-obturator route. Types of surgery that are not allowed in the study are abdominal prolapse surgery, conventional techniques like a Kelly plication, colpocleisis and the use of mini-slings.

### Randomization

Patients with SUI will be randomized through a website according to a computer-generated randomization sequence. The randomization sequence is computer generated with a block size of four. Stratified randomization is applied for centre and leading edge of the prolapse. Randomization will be 1:1 for concomitant surgery and prolapse surgery only. The data are web based registered. Women receive a case number at randomization to treat their data anonymously.

### CUPIDO II Study aims

The primary aim of the CUPIDO II study is to compare postoperative SUI in combined prolapse and anti-incontinence surgery or prolapse surgery only in patients with OSUI. Secondary aim is to determine whether concomitant surgery leads to more adverse events, like obstructive voiding symptoms and detrusor overactivity. Furthermore we want to evaluate the clinical value of a standardized redression test (on clinical examination or urodynamic investigation) to predict postoperative SUI. This could help to select patients that could benefit from concomitant surgery.

### Study design and setting

**Figure 2:**
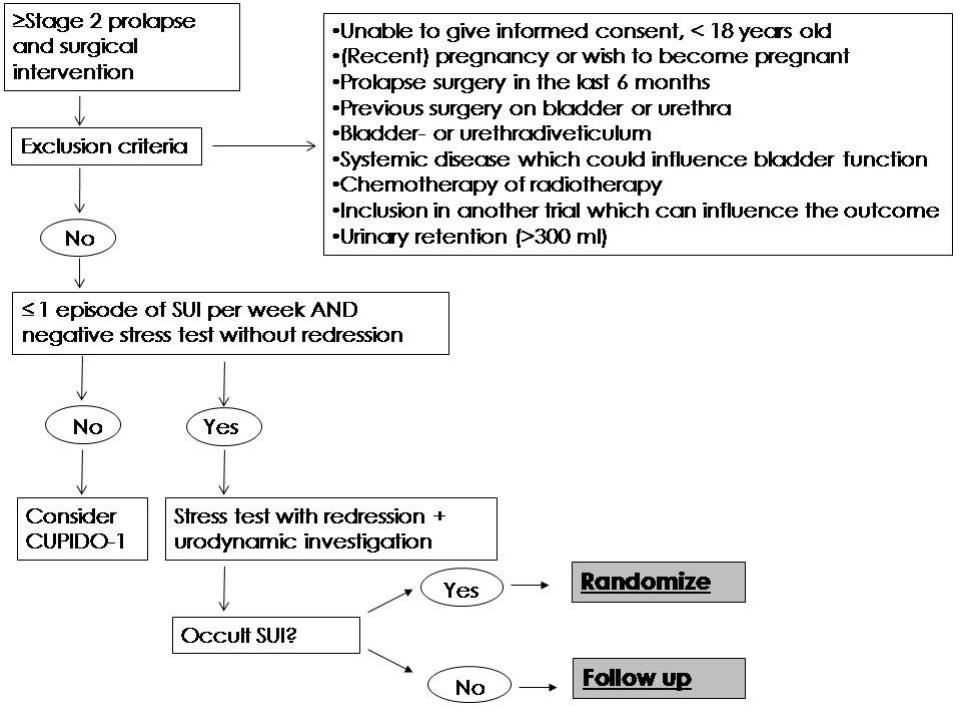
**Flowchart CUPIDO II**.

The CUPIDO II trial is a multicenter randomized controlled trial with a diagnostic cohort study. Women are enrolled with POP and no history of SUI and a negative cough test on examination. If OSUI is determined on clinical examination or urodynamic investigation, using a cough test while redressing the prolapse, these women are also randomized for concomitant surgery or prolapse surgery alone (CUPIDO II - randomisation). If no occult SUI is found, the risk of developing postoperative SUI is considered low. These women are not randomized and only prolapse surgery is performed (CUPIDO II - follow up). The follow up for these patients is similar to the women who were randomized in CUPIDO II, and is used to determine the need for urodynamic investigation in these patients, and the value of a standardized redression test. The outline of the CUPIDO II trial is shown in Figure 2.

### Determination and identification of eligible patients

Eligible patients will be selected by gynaecologists of all participating hospitals in the Netherlands (Table 1). All women with at least stage 2 POP in whom operative correction of the prolapse is considered will be asked for informed consent to participate the trial. Patients without SUI will have a stress test with redression of the prolapse, either on clinical examination or during urodynamic investigation. Patients with predominant urge incontinence are excluded. Women will also be excluded in case of previous incontinence surgery, recent or current pregnancy, wish for pregnancy in the future, history of bladder- or urethrasurgery, systemic disease which can affect the bladderfunction, chemo- or radiotherapy for cancer, chronic retention of the bladder (> 300 ml), and participation in another intervention study which can have influence on the findings in this trial (Table 3).

**Table 3:** Inclusion and exclusion criteria for CUPIDO II.

Inclusion criteria	Exclusion criteria
≥ 18 years old	Inability to give informed consent
≥ stage 2 prolapse	(Recent or planned) pregnancy
Intended surgery for prolapse	Previous anti-incontinence surgery
	Recent prolapse surgery (<6 months)
	Previous surgery to bladder or urethra or diverticulum
	Systemic disease which could influence bladder function
	Planned chemo- or radiotherapy for neoplasm
	Participation in another study which could influence the results
	Chronic urinary retention
	Cupido 2: SUI ≥ once a week OR positive cough test

### Interventions

Patients are randomized for concomitant surgery or prolapse surgery only. Prolapse surgery can be a vaginal hysterectomy or sacrospinous fixation, Manchester Fothergill operation, anterior colporrhaphy or mesh implantation, posterior colporrhaphy or mesh implantation and enterocele repair. Anti-incontinence surgery can be a midurethral sling, either using the retropubic route or the trans-obturator route. Types of surgery that are not allowed in the study are abdominal prolapse surgery, conventional techniques like a Kelly plication, colpocleisis and the use of mini-slings.

### Randomization

Patients with OSUI can be randomized after the redression test. The randomization sequence is computer generated with a block size of four. Stratified randomization is applied for centre and leading edge of the prolapse. Randomization will be 1:1 for concomitant surgery and prolapse surgery only. The data are web based registered. Women receive a case number at randomization to treat their data anonymously.

### Outcome measurements

The outcome measures are equal for both trials. Their primary outcome is absence of urinary (stress) incontinence and subsequent treatment for urinary (stress) incontinence 12 months after surgery. Secondary outcomes are absence of SUI at 6 months after surgery, anatomical results and repeated treatment for pelvic organ prolapse, disease specific and general quality of life, morbidity and quality of life, and quality adjusted life-years at 6 and 12 months postoperatively. We will also study general satisfaction and costs.

Patients in the studies will be followed from surgery until 12 months later. Follow up will consist of various elements. At inclusion the following data will be recorded:

1. History and clinical examination

2. 48 hour voiding diary

3. Dutch validated version of the Urinary Distress Inventory, a disease specific questionnaire comprising 17 questions, to assess the presence and experienced discomfort of pelvic floor problems. The UDI consists of 5 domains: discomfort/pain, urinary incontinence, overactive bladder, genital prolapse, and obstructive micturition. The total UDI score is defined as the average of the 5 domain scores, and can be used to assess cost effectiveness by measuring quality of life [20,21].

4. Short Form 36, with 36 questions on physical, mental and social health to assess generic quality of life [22].

5. EQ-5D, a disease non-specific quality of life questionnaire, to derive health utilities and the corresponding quality adjusted life years (QALYs) This is the change in quality of life induced by the treatment multiplied by the duration of treatment effect [23,24].

6. Health and Labour Questionnaire Short Form, to measure the impact of disease and treatment on absence from work, reduced productivity, unpaid labor production, and labor- related problems [25]

7. GIS question, a global impression of the prolapse and urinary complaints

8. Stress test with a bladder volume of at least 300 millilitres. The patient is instructed to cough four or five times while lying in lithotomy position. In case of a negative stress test, the test is repeated with redression of the prolapse. This is done by repositioning the cervix or vaginal vault using a cotton swab.

9. Post-voiding residual bladder volume measured by ultrasound

10. Examination of the prolapse using POP-Q

11. Examination of pelvic floor function

Follow up visits are planned with the patient's attending gynaecologist 6 weeks, 6 months and 12 months after surgery and exist of clinical examination including POP-Q, subjective full bladder stress test, residual bladder volume and detection of complications such as erosions. Along with the visits to the gynaecologist patients will receive the questionnaires and voiding diaries.

### Ethical considerations

Both studies have been approved by the Medical Ethical Committee of the Academic Medical Centre Amsterdam, December 2007. Full ethical approval for these studies has been obtained. (CMO number 07.17.1758). All participating centres obtained approval of their local Medical Ethical Committees.

### Economic evaluation

Cost effectiveness is evaluated using the costs per patient without of SUI at 12 months after surgery as the primary outcome measure. In addition, the cost utility is evaluated using the costs per quality adjusted life-year as the primary outcome measure. We aim to evaluate, from a societal perspective, whether concomitant surgery is more cost effective than prolapse surgery alone, with incontinence surgery in second stage if needed. We also want to evaluate whether the use of urodynamic investigation in continent women prior to surgery is more cost effective than non invasive methods to screen for OSUI. Incremental cost-effectiveness analyses will be performed and corresponding cost-effectiveness acceptability curves will be drawn for different values of society's willingness to pay to prevent one extra patient without SUI or to gain an extra quality adjusted life-year. Costs include direct medical costs, non-direct medical costs, and indirect costs of lost days at work and lost productivity while at work.

The needed data on resource use and health status will be gathered prospectively in case record forms and with patient questionnaires, in particularly, the Health and Labour Questionnaire short form and the EQ-5D.

Unit costs will be based on the Dutch 2004 guidelines for costing in health care research and indexed for base year 2008 using yearly general consumer price indices[26].

Quality adjusted life years will be derived from the observed EQ-5D health score profiles at baseline and during follow up by using available time trade-off based health valuation algorithms (and assuming each score profile to represent a patient's health status in-between the actual and the previous measurement [23,24].

### Sample size calculation

#### CUPIDO I trial

Of women with POP and predominant SUI 70% are expected to be cured of their SUI by prolapse surgery. A 20% increase in success rate is expected in concomitant surgery. For each arm 57 women are needed to prove the hypothesis with a power of 80% and one sided testing at 0.05. Taking into account 10% loss to follow up a total of 126 patients will be included in the study. We expect that 30% of the patients with POP will meet the criteria for the study.

#### CUPIDO II trial

For the group of women with occult SUI 73 are needed in each arm to prove a 15% difference in SUI 12 months postoperatively in concomitant surgery and prolapse surgery without midurethral sling (95% versus 80% continence respectively), with a power of 80% and α of 0.05. Taking into account 10% loss to follow up 160 patients will be randomized. We expect 60% of the patients with POP to meet the criteria for the study, and 50% of these patients to have OSUI. We will therefore include 320 women in the study to perform urodynamic investigation.

### Statistics

The analysis is performed according to the *intention to treat *principle. Data will be presented as numbers (percentage) for nominal variables or means (standard deviation) for interval variables. For interval variables, differences between the two groups will be analyzed with a Students t-test, proportions will be compared using the Chi-square test.

### Time plan

Inclusion for the Cupido study began in November 2007. The inclusion is planned to be finished in July 2010 for Cupido 1 and in March 2011 for Cupido 2. Patients are followed up to 12 months after surgery, so this will take until July 2011 and March 2012 for Cupido 1 and 2 respectively. The study is conducted in several Dutch centers assembled in the urogynaecology consortium. This consortium consists of Dutch centers cooperating in multicenter trials.

### Knowledge transfer

To date it is unknown whether prolapse surgery should be combined with a midurethral sling in patients with SUI or OSUI. These studies will provide an answer to the question if concomitant surgery is preferred above single prolapse surgery in women with SUI and OSUI. This study will be the starting point for a doctor's thesis. The outcomes of the study will be shared in different national and international scientific societies, and hopefully the results will be published in international scientific journals.

## Competing interests

None of the authors have competing interests arising from this research. The CUPIDO II trial has been awarded by a grant of the Dutch "Ohra Fonds".

## Authors' contributions

MD and JR designed the trial and were responsible for the development of the protocol. JR and HV are project leaders and have overall responsibility for the trial. AS and MP are responsible for the overall logistical aspects of the study, and AS drafted the paper. All authors have read and approved the final manuscript.

## Pre-publication history

The pre-publication history for this paper can be accessed here:

http://www.biomedcentral.com/1472-6874/10/16/prepub
